# University of Nebraska

**Published:** 1891-05

**Authors:** 


### EDITORIAL DEPARTMENT.

### NEBRASKA.

The Western States can teach their older sisters in the East, a great deal in the way of energy and of economy, by their interest in the advancement of agriculture and stock interests, and their closely allied supporter, veterinary medicine. They recognize, as should every State in union, the value of veterinary services. They appropriate annually for veterinary investigation and research, what New York or Pennsylvania, with a much heavier investment in live stock, refuses, even as a foundation for veterinary institutions, whose work is carried on practically as a labour of love. Every stone, however, to the establishment of veterinary medicine, means a stronger basis, and the prospect of growth, and we hail with pleasure, everything which aids the profession. We trust that the action, of the Board of Regents of the University of Nebraska, as given below will furnish an example to be followed, as it should be, by all of the States.

EXTRACT FROM REGENTS RECORD, UNIVERSITY OF NEBRASKA.

April 8, 1891.

"*Whereas*, the live stock interests of this state, represented by a special committee, and otherwise, request and urge renewal of investigations of diseases of domestic animals; and that Dr. F. S. Billings or some person of equal earnestness and ability be appointed to conduct and carry on such investigations, and

*Whereas*, this Board is convinced of the importance of said measures, and the magnitude of property interests involved, now, therefore,

*Be it resolved*, that this Board take steps to renew at the earliest practicable moment, such investigations; that Dr. F. S. Billings be employed for a period of one year from July 1, 1891, to conduct said investigations under the authority and direction of this Board; that for the purpose of covering the cost of said proposed investigations there is hereby appropriated from the Hatch Experiment Station fund, so-called, for the year commencing July 1, 1891, the sum of $10,050, to be applied as follows:

| | |
|---|---|
| Salary of Dr. Billings, - - - - | $3,600.00 |
| Salary of an assistant, - - - - | 1,200.00 |
| Salary of a Chemist, - - - - | 2,000.00 |
| Fitting up building, - - - - | 750.00 |
| Laboratory equipment, - - - | 500.00 |
| Incidental expenses, - - - - | 2,000.00 |
| | $10,050.00 |

That payment of salaries be made quarterly, or monthly if practicable, and that other expenditures contemplated be covered by duly approved vouchers as are other obligations of the experiment station, and the University :

*Resolved*, that the Fine Stock Breeders' Association be and are hereby requested to appoint a Conference Committee of not less than three prominent and experienced live-stock men, who shall observe, suggest and recommend concerning experiments and investigations and the character of the work of the investigator selected, and meet from time to time with the Board and confer thereon, and also use their influence through the State to advance and promote the service hereby contemplated."

Adopted.

J. S. Dales,
*Secretary of the Board of Regents,*
*University of Nebraska.*

